# Systematic evaluation of markers used for the identification of human induced pluripotent stem cells

**DOI:** 10.1242/bio.022111

**Published:** 2017-01-15

**Authors:** Sumitha Prameela Bharathan, Kannan Vrindavan Manian, Syed Mohammed Musheer Aalam, Dhavapriya Palani, Prashant Ajit Deshpande, Mankuzhy Damodaran Pratheesh, Alok Srivastava, Shaji Ramachandran Velayudhan

**Affiliations:** 1 Department of Haematology, Christian Medical College, Vellore, Tamil Nadu, India; 2 Centre for Stem Cell Research (Unit of InStem, Bengaluru), Christian Medical College Campus, Vellore, Tamil Nadu, India

**Keywords:** Reprogramming, Human induced pluripotent stem cells, Retroviral silencing, hiPSC morphology, Colony identification, Pluripotency

## Abstract

Low efficiency of somatic cell reprogramming and heterogeneity among human induced pluripotent stem cells (hiPSCs) demand extensive characterization of isolated clones before their use in downstream applications. By monitoring human fibroblasts undergoing reprogramming for their morphological changes and expression of fibroblast (CD13), pluripotency markers (SSEA-4 and TRA-1-60) and a retrovirally expressed red fluorescent protein (RV-RFP), we compared the efficiency of these features to identify bona fide hiPSC colonies. The co-expression kinetics of fibroblast and pluripotency markers in the cells being reprogrammed and the emerging colonies revealed the heterogeneity within SSEA-4^+^ and TRA-1-60^+^ cells, and the inadequacy of these commonly used pluripotency markers for the identification of bona fide hiPSC colonies. The characteristic morphological changes in the emerging hiPSC colonies derived from fibroblasts expressing RV-RFP showed a good correlation between hiPSC morphology acquisition and silencing of RV-RFP and facilitated the easy identification of hiPSCs. The kinetics of retroviral silencing and pluripotency marker expression in emerging colonies suggested that combining both these markers could demarcate the stages of reprogramming with better precision than with pluripotency markers alone. Our results clearly demonstrate that the pluripotency markers that are routinely analyzed for the characterization of established iPSC colonies are not suitable for the isolation of pluripotent cells in the early stages of reprogramming, and silencing of retrovirally expressed reporter genes helps in the identification of colonies that have attained a pluripotent state and the morphology of human embryonic stem cells (hESCs).

## Abstract (precis)

**Summary:** The use of hESC-like morphology, retroviral transgene silencing and temporal expression of pluripotency markers are compared as methods to aid in the identification of hiPSC clones.

## INTRODUCTION

Human induced pluripotent stem cells (hiPSCs) resemble human embryonic stem cells (hESCs) at molecular and functional levels. As individual specific hiPSCs can be generated, they are considered to be better than hESCs for disease modeling, drug screening, and regenerative medicine (Robinton and Daley, 2012; Takahashi et al*.*, 2007). hiPSCs have been successfully derived from different somatic cell types by expressing various combinations of reprogramming factors (Bayart and Cohen-Haguenauer, 2013; González et al*.*, 2011). One of the limitations of the current reprogramming strategies is the low efficiency in pluripotency induction resulting in the generation of a very few reprogrammed hiPSC colonies compared to the starting number of donor cells (Bayart and Cohen-Haguenauer, 2013). This low efficiency leads to morphological and molecular heterogeneity among the generated colonies (Chan et al*.*, 2009; Liang and Zhang, 2013), which makes the identification and isolation of bona fide hiPSC clones from a reprogramming dish tedious and necessitates extensive characterization of the isolated clones for their pluripotency.

Although the ESC-like morphology of iPSCs has been used for their isolation from a reprogramming dish, several studies have shown that the partially reprogrammed mouse and human iPSCs do not differ morphologically from fully reprogrammed clones (Okita et al*.*, 2007). Fluorescent reporter genes knocked into the endogenous loci of the core pluripotency genes have significantly improved the efficiency in the derivation of iPSCs (Hotta and Ellis, 2008; Okita et al*.*, 2007; Takahashi and Yamanaka, 2006; Wernig et al*.*, 2007; Yu et al*.*, 2007). Sorting of cells that express pluripotency surface markers, without additional genetic modifications of donor cells to express the reporter genes, has also been used for enriching the pluripotent cell population. Combining positive and negative surface markers also allows the selection and expansion of iPSCs with a relative reduction in the effort required to culture partially reprogrammed iPSC clones (Abujarour et al*.*, 2013; Dick et al*.*, 2011; Kahler et al*.*, 2013; Quintanilla et al*.*, 2014; Valamehr et al*.*, 2012). However, such enrichment methods generate heterogeneous colonies that lack clonal identity as they consist of cells originated from different donor cells. Additionally, these protocols involve the single-cell culture of hiPSCs that require modified culture conditions, and the clones generated from single cells often are at increased risk of karyotypic abnormalities (Valamehr et al*.*, 2012). Though live cell imaging of surface markers could be used for isolation of hiPSC clones (Chan et al*.*, 2009; Lowry et al*.*, 2008; Polak et al*.*, 2012), these markers are even expressed by transgene-dependent partially reprogrammed cells, and a combination of additional intracellular markers is required to distinguish the fully reprogrammed state from the partially reprogrammed state in the isolated clones (Abujarour et al*.*, 2013; Chan et al*.*, 2009; Tanaka et al*.*, 2015). The ability of pluripotent stem cells to silence the transgenes expressed from retroviral vectors has also been explored as a marker for identification and isolation of pluripotent clones (Chan et al*.*, 2009; Hotta and Ellis, 2008; Nakagawa et al*.*, 2008). Despite being a reliable indicator of transgene independence in iPSC clones, retroviral transgene silencing has not been extensively used as a marker for identification of hiPSCs.

We systematically analyzed expression of fibroblast and pluripotency markers to study the heterogeneity of reprogramming cells. We also evaluated the silencing of retroviral fluorescent protein and morphology of the cells at different stages of reprogramming and the emerging colonies to assess their ability to identify bona fide pluripotent cells and colonies in the early and later stages of reprogramming.

## RESULTS

### The expression pattern of fibroblast and pluripotent cell markers in reprogramming cells and emerging hiPSC colonies

Several studies have been carried out to correlate expression patterns of individual surface markers with the transformation of cells during human somatic cell reprogramming, as a means to enrich the population of pluripotent stem cells in order to increase the efficiency of reprogramming and for studying the mechanisms of reprogramming (Abujarour et al*.*, 2013; Chan et al*.*, 2009; Hotta et al*.*, 2009; Kahler et al*.*, 2013; Tanaka et al*.*, 2015). However, a systematic analysis of their temporal expression to assess their reliability for the isolation of reprogramming intermediates and pluripotent clones has been lacking. Therefore, we monitored the expression kinetics of surface markers of fibroblasts (CD13) and pluripotent cells (SSEA4 and TRA-1-60) in the reprogramming cells before and after the formation of colonies to find their coexpression pattern.

**Fig. 1.**
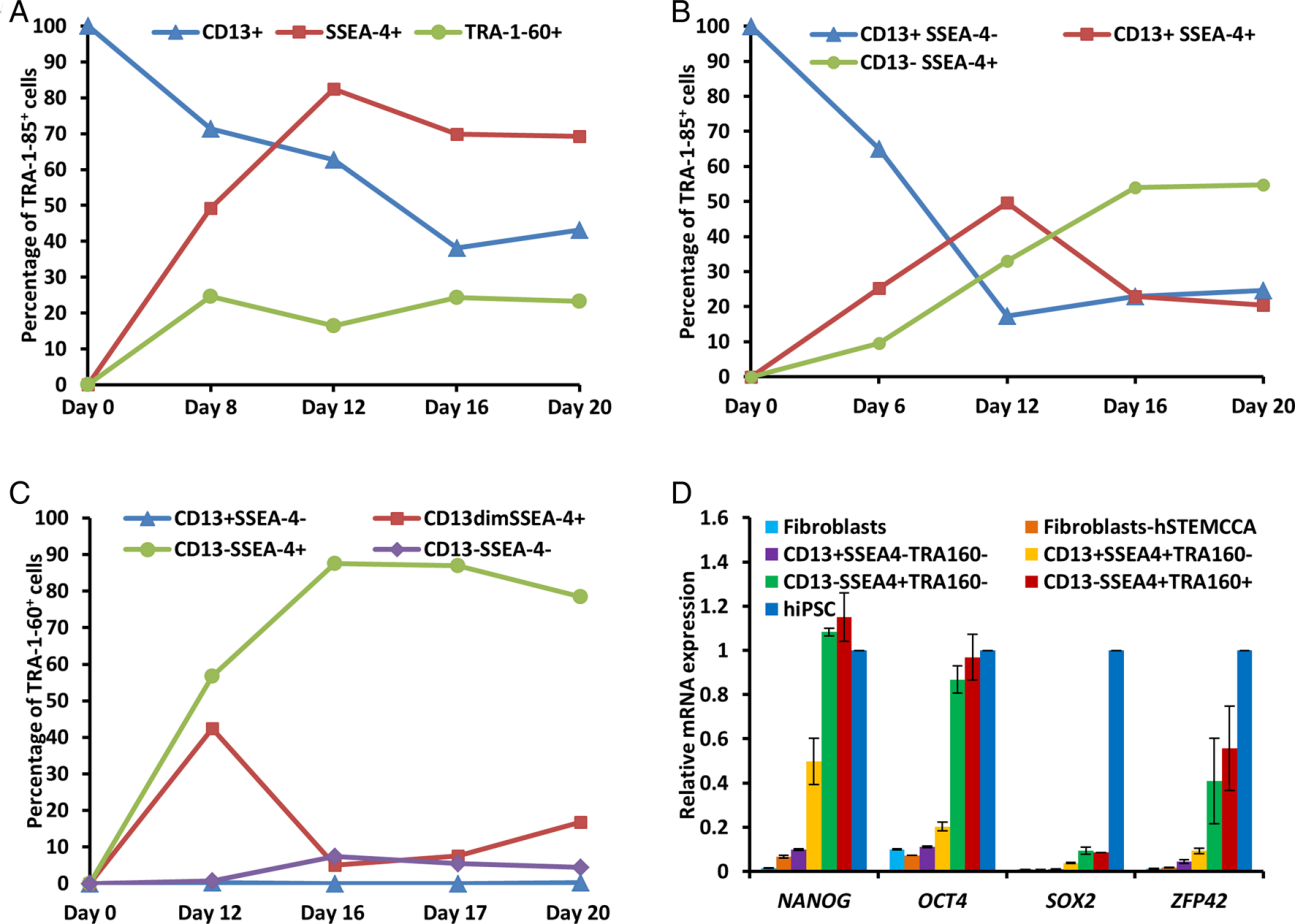
**Analysis by flow cytometry of fibroblast (CD13) and pluripotency (SSEA-4 and TRA-1-60) markers during reprogramming of hADFs.** (A) Percentages of CD13^+^, SSEA-4^+^ and TRA-1-60^+^ cells on different days of reprogramming in the TRA-1-85^+^ population of cells. (B,C) Percentages of the cells that co-express CD13 and SSEA-4 in the (B) TRA-1-85^+^ population of cells and in the (C) TRA-1-60^+^ fraction of cells. (D) Real-time PCR analysis of mRNA levels of pluripotency markers, *NANOG*, *OCT4*, *SOX2* and *ZFP42* in hADFs, in the cells 6 days after transducing with hSTEMCCA lentiviruses, and the cell fractions sorted by flow cytometry based on the expression of CD13, SSEA-4 and TRA-1-60 (*n*=2). The fold-changes were calculated relative to expression levels in hiPSCs. Data represented as mean±s.d.

Following transduction of dermal fibroblasts with hSTEMCCA lentiviral vectors, the reprogramming cells were analyzed on days 6, 8, 12, 16, 17 and 20 for the expression of CD13, SSEA-4 and TRA-1-60, within the cells expressing TRA-1-85, a pan-human cell maker used for avoiding the interference of feeder cells. The percentage of CD13^+^ cells reduced during reprogramming and the percentage of SSEA-4^+^ and TRA-1-60^+^ cells increased (Fig. 1A; Fig. S1a). However, we found that most of the reprogramming cells (70-80%) achieved SSEA-4 expression by day 12. But, a large fraction of the SSEA-4^+^ cells was also CD13^+^, 60% in the second week and 30% in the third week. The ratios of the percentages of CD13^+^SSEA-4^−^, CD13^+^SSEA-4^+^ and CD13^−^SSEA-4^+^ cells were, 65:25:10 on day 6, 17:50:33 on day 12, 23:23:54 on day 16 and 25:20:55 on day 20 (Fig. 1B; Fig. S1b). Analysis of TRA-1-60 expression showed that ∼20% of the reprogramming cells were TRA-1-60^+^ on day 8, and its level remained nearly constant till day 20 (Fig. 1A). Almost all TRA-1-60^+^ cells (>95%) were CD13^−^SSEA-4^+^ throughout the reprogramming process, and only a small fraction of CD13^dim^SSEA-4^+^TRA-1-60^+^ cells (<5%) was observed in the first week of reprogramming (Fig. 1C). This suggested that TRA-1-60 expression is initiated in the SSEA-4^+^ cells after the somatic cell gene silencing is almost complete. We sorted reprogramming cells by FACS based on the expression of CD13, SSEA-4 and TRA-1-60 and different fractions were analyzed for the expression of late stage pluripotency genes, *NANOG*, *OCT-4*, *SOX2* and *ZFP42*. Their levels increased steadily from CD13^+^SSEA-4^+^TRA-1-60^−^ to CD13^−^SSEA-4^+^TRA-1-60^−^ to CD13^−^SSEA-4^+^TRA-1-60^+^ cells (Fig. 1D). Based on this data and the change in the relative percentage of cells expressing a different combination of markers (Fig. 1A-C) it could be proposed that, in a successful reprogramming, the sequence of states that CD13^+^SSEA-4^−^TRA-1-60^−^ fibroblasts transit through are CD13^+^SSEA-4^+^TRA-1-60^−^, CD13^dim^SSEA-4^+^TRA-1-60^+,^ and CD13^−^SSEA-4^+^TRA-1-60^+^, and the temporal analysis of these markers allows isolation of cell fractions at different stages of reprogramming. The cells that fail to transit through all these stages remain incompletely reprogrammed as CD13^+^SSEA-4^−^TRA-1-60^−^, CD13^+^SSEA-4^+^TRA-1-60^−^, CD13^−^SSEA-4^+^TRA-1-60^−^, CD13^−^SSEA-4^−^TRA-1-60^−^ and CD13^−^SSEA-4^−^TRA-1-60^+^.

**Fig. 2.**
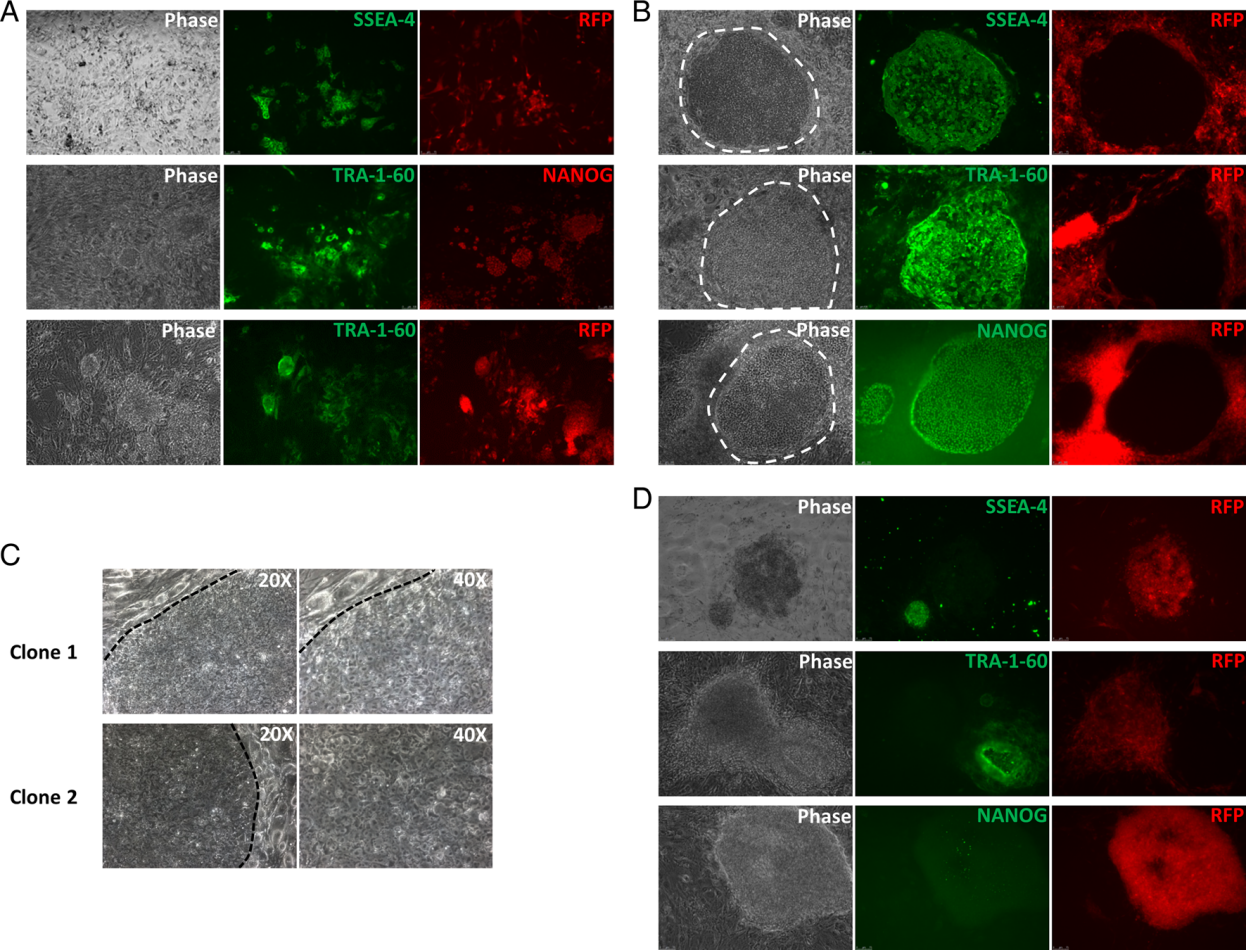
**Analysis of morphology, pluripotency marker expression and transgene silencing in the colonies emerging during reprogramming.** (A) Expression of SSEA-4 and RV-RFP (upper panel), TRA-1-60 and NANOG (middle panel) and TRA-1-60 and RV-RFP (lower panel) in the cell clusters/colonies on day 9 of reprogramming showing the initiation of pluripotency marker expression before the cells achieve hESC-like morphology and transgene silencing. (B) Expression of the pluripotency markers (SSEA-4, TRA-1-60 and NANOG) and RV-RFP silencing in the colonies on day 16 of reprogramming. The emerging hiPSC colonies showed characteristic hESC-like morphology and retroviral transgene silencing allowing their easy identification. (C) Higher magnification images of RV-RFP^−^ hiPSC colonies showing their hESC-like morphology – flat appearance, defined boundary and high nuclear-to-cytoplasmic ratio. (D) Non-hESC like RFP^+^ colonies which lacked the expression of pluripotency markers on day 16. All images are at 10× magnification, unless otherwise indicated. The broken lines show the characteristic boundaries of the emerging hiPSC colonies on the feeder cells.

When the emerging colonies were analyzed for the expression of pluripotency markers, SSEA-4, TRA-1-60 and NANOG, on different days of reprogramming by immunofluorescence, the trend in their expression kinetics was similar to that obtained by flow cytometric analysis of reprogramming cells. On day 9, small cell clusters or colonies made up of 20-60 tightly packed cells were visible. Although they lacked hESC morphology, most of them expressed SSEA-4 (Fig. 2A, upper panel), but only ∼10% of the SSEA-4^+^ cell clusters expressed TRA-1-60. All these TRA-1-60^+^ colonies also expressed NANOG from day 9 of reprogramming (Fig. 2A, middle panel) suggesting that the reprogramming cells achieve the CD13^−^SSEA-4^+^TRA-1-60^+^NANOG^+^ pluripotent state in the very early days of reprogramming (less than 10 days) even before hiPSC colonies attain hESC-like morphology. By the third week of reprogramming (day 16), as the colony size increased, their morphologies became evident, and many large hiPSC colonies expressed SSEA-4, TRA-1-60 and NANOG (Fig. 2B). The reprogramming efficiency that was estimated based on the number of TRA-1-60^+^ colonies remained constant (∼ 0.1%) in the second and the third weeks of reprogramming.

Taken together, our results showed that a large number of reprogramming cells achieve SSEA-4 and TRA-1-60 expression from early days, although the efficiency of the formation of hiPSC colonies is extremely low (<1%). SSEA-4^+^ cells are molecularly heterogeneous throughout reprogramming, which was evident from their temporal changes in the expression of CD13, whereas TRA-1-60^+^ cells were relatively less heterogeneous. Since only a small fraction of the cells expressing SSEA-4 and TRA-1-60 markers represents pluripotent cells, there is a demand for markers that can further define the molecular states within these cells for a more reliable identification of pluripotent cells and stages of reprogramming.

### The correlation between retroviral transgene silencing, pluripotency marker expression and morphology of the emerging hiPSC colonies

In reprogramming, silencing of the reprogramming factor transgenes marks exogenous factor independence for the maintenance of pluripotency of hiPSCs (Aasen et al*.*, 2008; Okita et al*.*, 2007; Stadtfeld et al*.*, 2008; Takahashi et al*.*, 2007). Retroviral transgenes (RV-Tg) driven by long terminal repeats (LTRs) are effectively silenced in pluripotent stem cells (Hotta and Ellis, 2008) and silencing of retrovirally expressed fluorescent proteins has been correlated with pluripotency (Chan et al*.*, 2009; Hotta et al*.*, 2009). However, retroviral transgene silencing has not been correlated systematically with the expression of other pluripotency markers to understand the kinetics of transgene silencing and pluripotency induction in successful reprogramming events. Morphological features of the hiPSC colonies have been widely employed for their identification from a reprogramming dish and, therefore, we also looked at the correlation between RV-RFP and pluripotency marker expression with the morphology of the emerging colonies.

We reprogrammed fibroblasts transduced with retroviruses to express RFP along with OSKM, and the cells were monitored for the expression of the retroviral RFP (RV-RFP), NANOG, SSEA-4 and TRA-1-60 in the emerging clones. On day 11, 65-70% of the SSEA-4^+^ colonies were TRA-1-60^+^, but ∼65% of the TRA-1-60^+^ colonies were RV-RFP^+^ (Fig. 2A, lower panel). On day 18, about 85% of the TRA-1-60^+^ colonies were RFP^−^. The reprogramming efficiency estimated based on the total number of TRA-1-60^+^ colonies was <0.1%, and it remained the same in the second and third weeks of reprogramming.

Monitoring of the growth features and the morphology of the emerging colonies showed that as RFP^−^ colonies emerged they dislodged the feeder cells around them radially, forming a symmetric patch of RFP^−^ cells surrounded by RFP^+^ cells and they expressed NANOG, SSEA-4 and TRA-1-60 (Fig. 2B). The close microscopic observation of RFP^−^ colonies showed that they possessed typical hESC morphology – flat colonies containing closely packed cells with increased nuclear-to-cytoplasmic ratio (Fig. 2C). This morphology was similar to a well-established hiPSC line that was maintained in our laboratory (BC1-hiPSC line; a gift from Linzhao Cheng, John Hopkins Medicine, Baltimore, MD, USA) (data not shown). All the colonies without hESC morphology were RFP^+^ when they were analyzed in the third week of reprogramming, and they lacked the expression of SSEA-4, TRA-1-60 and NANOG (Fig. 2D).

**Fig. 3.**
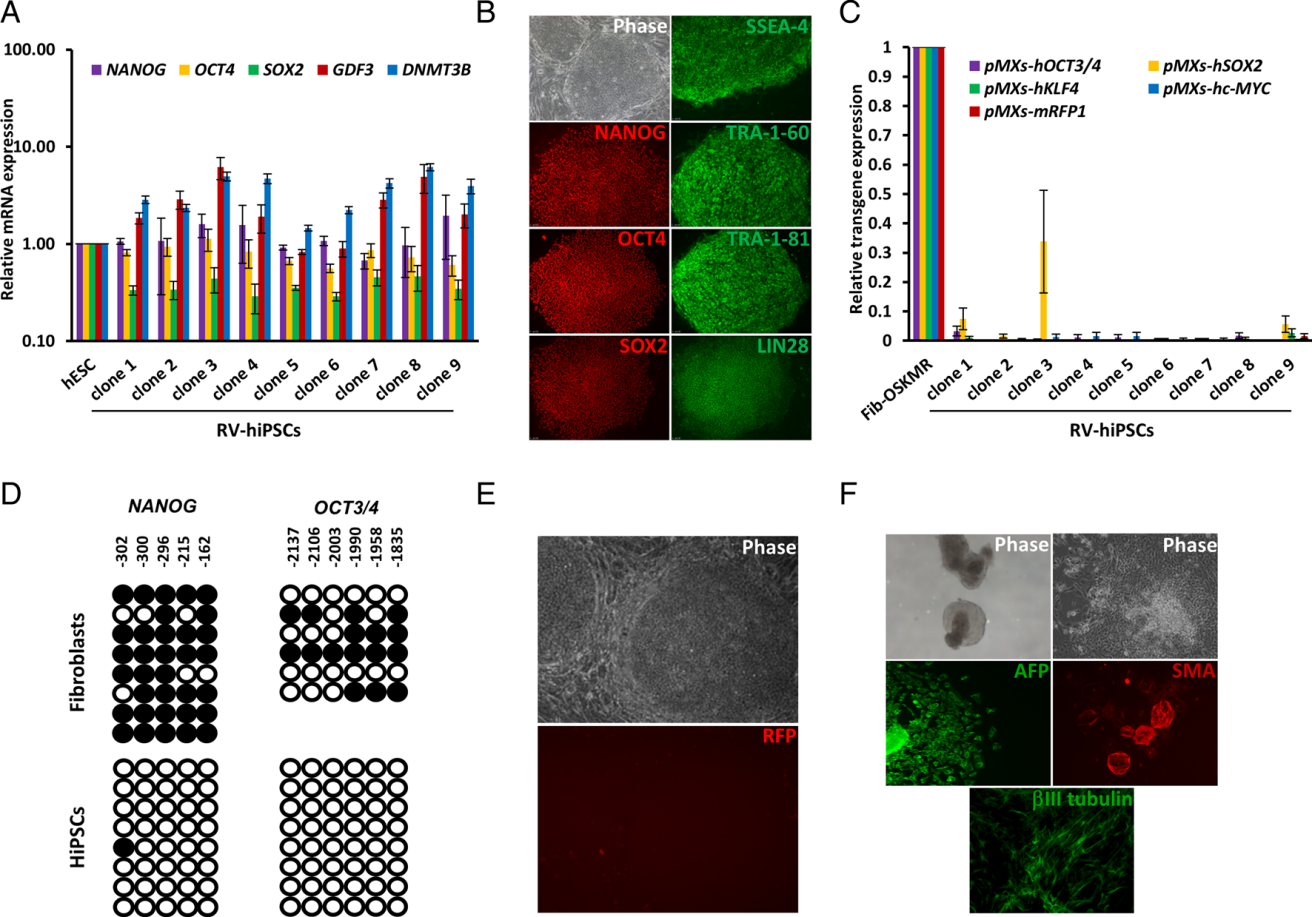
**Characterisation of RV-hiPSC clones isolated based RV-Tg silencing and hESC-like morphology.** (A) Real time PCR analysis of pluripotency markers in the isolated RV-hiPSC clones. The fold-change was calculated relative to the expression levels in hESCs (*n*=2). (B) Immunofluorescence analysis of pluripotency markers in the isolated clones. (C) Real-time PCR analysis of expression of retroviral transgenes in the clones (*n*=2). The fold-changes were calculated relative to the expression levels in fibroblast transduced with OSKMR (Fib-OSKMR). (D) Bisulfite sequencing results of *OCT4* and *NANOG* promoters in fibroblasts and established RV-hiPSC clones showing hypomethylation of these regions. (E) Microscopic image of an established RV-hiPSC clone confirming the stable silencing of transgenes throughout the culture. (F) *In vitro* differentiation of established hiPSC clones. Data from a representative clone is shown. hiPSCs formed cystic embryoid bodies (EBs) in suspension culture and these EBs were differentiated further in an adherent culture into the cells expressing markers characteristic of three germ layers – endoderm (α-feto protein, AFP), mesoderm (α-smooth muscle actin, SMA) and ectoderm (βIII-Tubulin). All images are at 10× magnification. Data represented as mean±s.d.

We established nine retrovirally generated hiPSC lines (RV-hiPSCs) from the RFP^−^ colonies with morphological features described above. These cell lines maintained high expression of pluripotency markers (Fig. 3A,B), silencing of all the transgenes (Fig. 3C,E) and hypomethylation of *OCT4* and *NANOG* promoters (Fig. 3D) even after 15-20 passages. We expanded three of these clones in long-term culture (>50 passages) without the loss of morphology and the expression of pluripotency markers. The pluripotency of one of these clones was further confirmed by its *in vitro* differentiation potential (Fig. 3F).

Taken together, by observing the emerging hiPSC colonies derived from RV-RFP-transduced fibroblasts, we identified characteristic temporal morphology changes that successfully reprogramming cells undergo and concluded that RV-RFP silencing could be employed as a marker to identify the hiPSC colonies with the morphology and pluripotency levels of hESCs. The RV-RFP expression status could partly explain the molecular difference between the TRA-1-60^+^ cells in the early and late stages of reprogramming.

### Isolation of integration-free hiPSC clones based on morphology

**Fig. 4.**
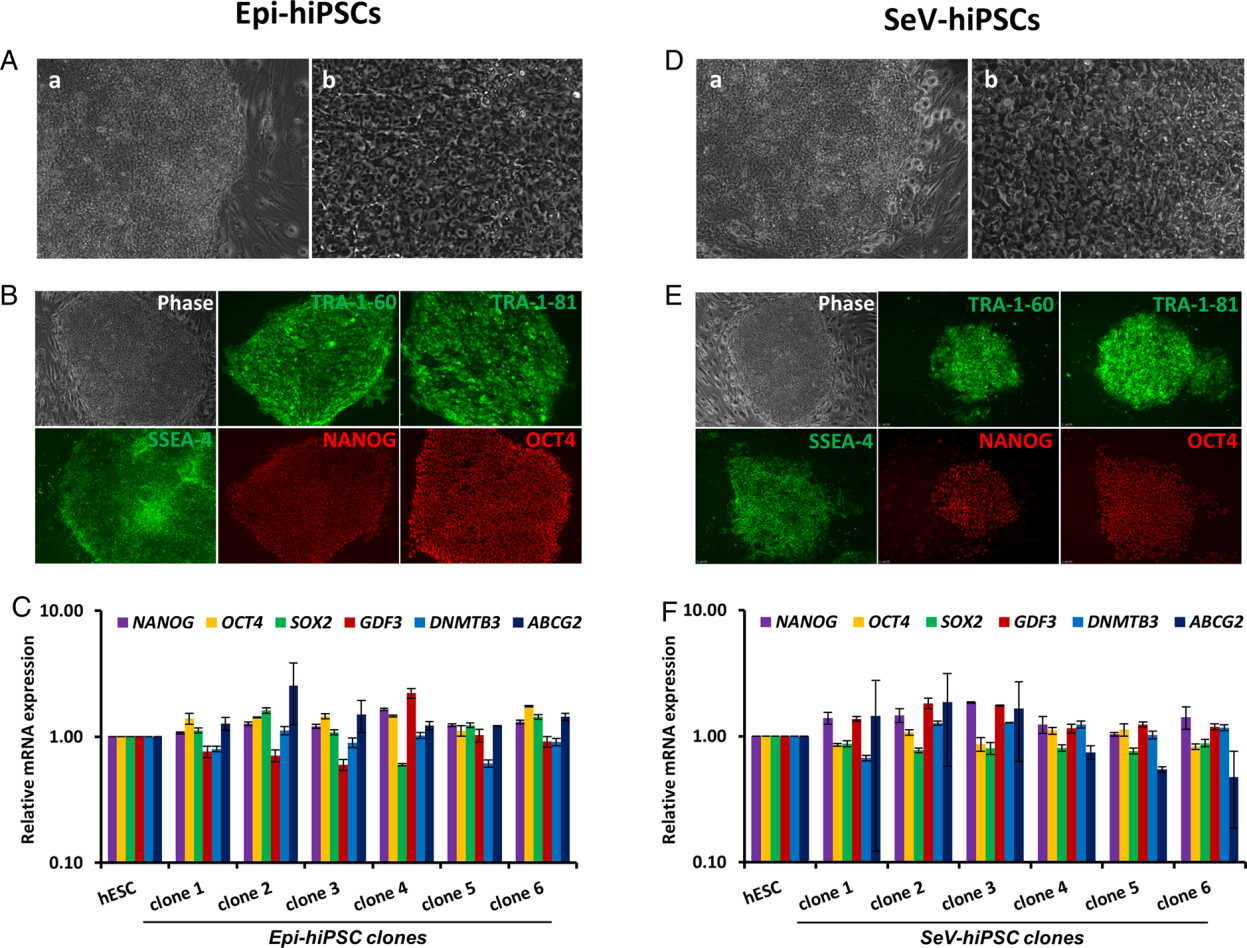
**Morphology-based isolation of hiPSCs generates clones with high levels of pluripotency.** hiPSC clones were generated using episomal (Epi) plasmids or Sendai Virus (SeV) to express the reprogramming factors. (A) Morphology of a representative emerging hiPSC colony generated by episomal plasmids and (D) by Sendai viruses. Images in (a) lower (10×) and (b) higher (20×) magnifications are shown to represent the defined boundaries and the increased nuclear-to-cytoplasmic ratio of the emerging colonies. (B) Morphology and immunofluorescence analysis of pluripotency markers in an established Epi-hiPSC line and (E) in an established SeV-hiPSC line. Images are at 10× magnification. (C) Real time PCR analysis of pluripotency markers in Epi-hiPSC lines and (F) in SeV-hiPSC lines (*n*=2). The fold-changes were calculated relative to the expression levels in hESCs. Data represented as mean±s.d.

After establishing the correlation between the morphology and RV-RFP silencing of hiPSC colonies, we decided to generate integration-free hiPSCs from the colonies isolated from the reprogramming dish based on morphology alone. We used two non-integrative vector systems to deliver reprogramming factors into fibroblasts cells; oriP/EBNA1-based episomal plasmids to express *OCT4*, *SOX2*, *KLF-4*, *L-MYC*, *LIN28* and *p53*-shRNA (Okita et al*.*, 2011) and Sendai viruses (SeV) to express *OCT4*, *SOX2*, *KLF-4* and *c-MYC* (Ban et al*.*, 2011). Using the episomal plasmids, we obtained a reprogramming efficiency of 0.01% estimated based on TRA-1-60 expression and acquisition of hESC-like morphology in colonies. We isolated six clones that had hiPSC morphology (Fig. 4A), and all the colonies showed consistent high-level expression of pluripotency markers over 10 passages (Fig. 4B,C). With SeV vectors we observed a reprogramming efficiency of 0.2% estimated based on TRA-1-60 expression and hESC-like morphology in colonies. The colonies with emerging hiPSC morphology were visible from the beginning of the third week (day 16) after transduction and, in the fourth week of reprogramming, nine colonies were isolated based on their hESC-like morphology (Fig. 4D) and six of them could be established as hiPSC lines. They maintained hESC-like morphology and showed a high-level expression of all the pluripotency markers (Fig. 4E,F).

These results showed that the laboratories that gained experience in the morphology of hiPSC colonies could derive highly pluripotent hiPSCs lines without the use of any additional markers. The morphology-based isolation is important for the generation of footprint-free hiPSCs using non-integrative reprogramming strategies involving episomal or SeV vectors.

### hiPSCs isolated based on morphology showed consistent *in vivo* differentiation potential

**Fig. 5.**
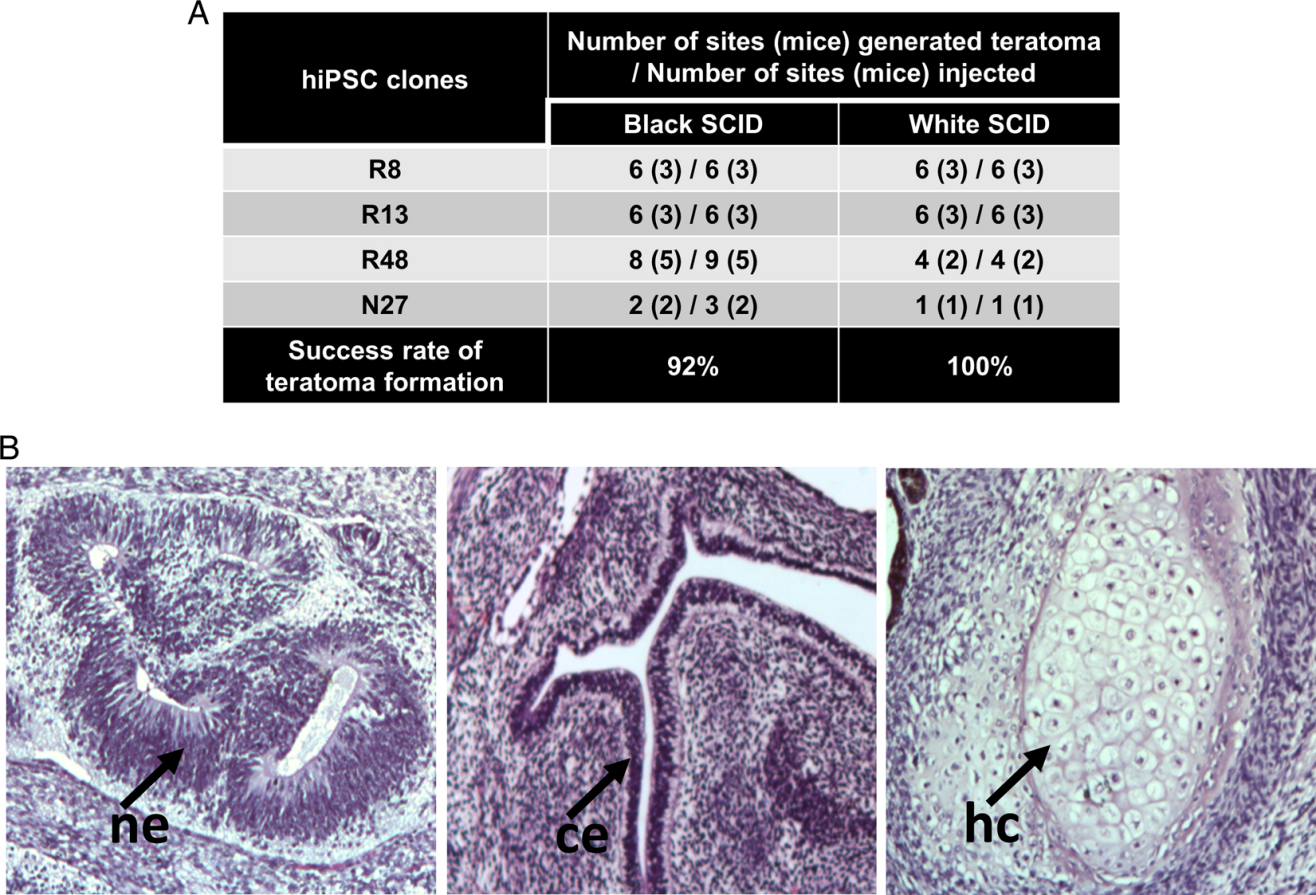
**hiPSC clones isolated based on morphology with and without using retroviral silencing as a marker generates high grade teratomas.** (A) Table showing the outcome of teratoma assays performed in black SCID and white SCID mice. R8, R13 and R48 are two RV-hiPSC lines and N27 is an Epi-hiPSC line. (B) Hematoxylin and eosin staining of formalin fixed teratoma sections showing tissues of all the three germ layers. Representative images are shown. ne, neuroepithelium (ectoderm); hc, hyaline cartilage (mesoderm) and ce, columnar epithelium (endoderm). Images are at 20× magnification.

For further confirmation of the pluripotency of hiPSC clones isolated based on their morphology, with or without the transgene silencing, their *in vivo* differentiation potential was tested by teratoma formation. Since the efficiency of teratoma formation depends on multiple factors like pluripotency level of the cells, the sites of injection and the host immunophenotype (Ozolek and Castro, 2011) we performed the teratoma assay in two strains of SCID mice, B6.CB17-*Prkdc^scid^*^/^SzJ (black SCID) or CB17/Icr-*Prkdc^scid^*/IcrIcoCrl (white SCID). To favor teratoma formation, the cells were injected with Matrigel and Collagen I intramuscularly into the hind limbs of 4-7-week-old mice, using a previously described protocol (Park et al*.*, 2008a). We performed teratoma assay with five hiPSC lines (three RV-hiPSC lines, one SV-hiPSC line and one Epi-hiPSC line), which were isolated based on the morphology and subsequently confirmed to have a high-level expression of pluripotency markers (Fig. 5A). All four lines formed teratomas constituting cells representing endoderm, mesoderm and ectoderm in 8 to 14 weeks (Fig. 5B). Both black and white SCID mice showed similar efficiencies in teratoma formation; 92% (22/24 sites) and 100% (17/17 sites), respectively (Fig. 5A). When hiPSCs suspended in hiPSC basal culture medium were injected without the use of Matrigel and Collagen I (Takahashi et al*.*, 2007), the efficiency of teratoma formation was very low (>10%; data not shown).

Thus, the high efficiency of teratoma formation by hiPSC clones derived in our laboratory confirmed that the clones isolated based on characteristic morphological features that we described here guarantees the isolation of bona fide hiPSC clones.

## DISCUSSION

The significant heterogeneity in the colonies generated during reprogramming makes the derivation of bona fide hiPSC lines that are suitable for downstream applications tedious, expensive and time-consuming. The heterogeneity of hiPSCs has been attributed to cell-to-cell genetic variations in the donor cells (Liang and Zhang, 2013), persistent expression of somatic cell­-specific genes (Kim et al*.*, 2011), residual expression of the reprogramming factors (Okada and Yoneda, 2011) and the low efficiency of the process to complete reprogramming (Raya et al*.*, 2009). Several studies have shown that the isolated hiPSC clones have differences in the expression of pluripotency markers (Chan et al*.*, 2009; Liang and Zhang, 2013) and those with the same levels of pluripotency marker expression had different abilities in lineage differentiation (Kim et al*.*, 2011; Polo et al*.*, 2010).

The iPSC enrichment and isolation criteria widely employed are still based on observable features of the colonies, like morphology and pluripotency marker expression (Chan et al*.*, 2009; Park et al., 2008b; Takahashi et al*.*, 2007). A side-by-side comparison of the efficiency of these methods to identify true iPSCs helps in assessing their reliability in isolating hiPSC clones and ability in defining heterogeneity among the colonies generated. When we observed the reprogramming cells as well as the emerging colonies throughout the reprogramming process, valuable insights were obtained in hESC-like morphology acquisition, pluripotency marker expression, and retroviral transgene silencing in the context of pluripotency induction.

Although the reprogramming process has several barriers that cause a severe reduction in the efficiency in the generation of hiPSC colonies, the shutdown of fibroblast markers and the activation of an early pluripotency marker, SSEA-4, occur in the majority of the reprogramming cells. The findings that a large number of SSEA4^+^ cells co-express a fibroblast marker in the early and later stages of reprogramming, and that ∼20% of the reprogramming cells are TRA-1-60^+^ although the reprogramming efficiency is only 0.1%, suggest that both SSEA-4^+^ and TRA-1-60^+^ are molecularly heterogeneous at different levels. The extent of heterogeneity in the SSEA-4^+^ and TRA-1-60^+^ cells that we observed has not been documented before. The TRA-1-60^+^ cells are mostly transgene-dependent in the early stages and independent in the late stages of reprogramming. Our study highlighted the significance of each of these markers in defining the stages of pluripotency induction and identification of bona fide hiPSC colonies during fibroblast reprogramming. The downregulation of fibroblast marker and induction of SSEA-4 occur in the early stages, induction of TRA-1-60 in the intermediate stages and silencing of the retroviral transgene in the late stages of reprogramming. Being a more reliable pluripotency marker, combining RV-Tg reporter with expression of CD13, SSEA-4 and TRA-1-60 will be more effective in demarcating the stages of reprogramming. Analysis of the cells belonging to reprogramming states represented by these four markers will help in understanding the sequential molecular events that occur during reprogramming and the major barriers involved, which will further aid in the development of more efficient strategies to improve the reprogramming efficiency.

By routine monitoring of RV-RFP expression of the emerging colonies, we identified characteristic morphological changes of the bona fide hiPSC colonies. Unlike SSEA-4 and TRA-1-60 that showed significant heterogeneity and a lack of correlation with true pluripotency and morphology of the emerging hiPSC colonies, there was a significant correlation between RV-Tg silencing and hESC-like morphology and pluripotency of these colonies. The expression of NANOG, a definitive marker of pluripotency by RV-RFP^−^ hESC-like colonies, and the generation of stable hiPSC lines that are capable of tri-lineage differentiation *in vitro* and *in vivo* confirmed that silencing of a retroviral LTR-driven fluorescent marker helps in the identification of the successfully reprogrammed hiPSC colonies from the heterogeneous colonies in the reprogramming dish. In the emerging hESC-like colonies, retroviral transgene silencing was found to be a more reliable hiPSC identification marker than SSEA-4 and TRA-1-60.

Monitoring RV-Tg expression is an extremely useful strategy for the isolation of RV-Tg-silenced hiPSC colonies by the laboratories that lack the expertise in the morphology-based isolation of hiPSCs, and this overcomes a major challenge involved in this identification method. By successfully deriving pluripotent hiPSC lines from fibroblasts reprogrammed with integrative and non-integrative vectors, we found that the morphology of hiPSCs is the best criterion to isolate highly pluripotent hiPSC colonies. We highlight the importance of following the morphological changes of the emerging hiPSCs that are easily observable on routine monitoring without the requirement of staining them for pluripotency markers. Due to the strong correlation between morphology and pluripotency, the need for genetic modification of donor cells, FACS/MACS­-based enrichment of the reprogramming cells and the single cell culture of hiPSCs can be avoided and bona fide hiPSC lines can be derived by carefully employing morphology­-based isolation of a few (two to three) colonies from the reprogramming dish.

The reliability of hESC-like morphology acquisition in emerging colonies during reprogramming to identify and isolate bona fide hiPSC colonies capable of teratoma formation is highlighted in this study. Previous studies have showed that the efficiency of teratoma formation of hESCs was 100% with kidney capsule injections, and 60% for intratesticular, 33% for subcutaneous and 12.5% for intramuscular injections (Prokhorova et al*.*, 2009). For hiPSCs, the subcutaneous injection was reported to give very low efficiencies in teratoma formation, and intratesticular injection showed up to 80% efficiency (Ohnuki et al*.*, 2009). One important finding in our study was the efficient teratoma formation that was observed after subcutaneous injection of hiPSCs. The extremely high success rate that was observed may be due to the protocol that was adapted for our experiments.

## MATERIALS AND METHODS

### Cell culture

PlatE (Cell Biolabs), HEK293T (ATCC), and SNL cells (a gift from Allan Bradley, Sanger Institute, Cambridge, UK) were cultured in Dulbecco's Modified Eagle's Medium (DMEM) with 10% fetal bovine serum (FBS), 2 mM L-glutamine and antibiotics, 50 U/ml penicillin and 50 μg/ml streptomycin. Human adult dermal fibroblasts (hADFs, Cascade Biologicals) were grown in alpha­-modified minimum essential medium (α-MEM) with 20% FBS, 2 mM L-glutamine and the antibiotics. Reprogramming was carried out with the hiPSC medium containing DMEM-F12 with 20% knockout serum replacement (KOSR), 2 mM L-GlutaMAX, 0.1 mM minimal essential medium–nonessential amino acids (MEM-NEAA) solution, 0.11 mM β-mercaptoethanol, 10 ng/ml basic fibroblast growth factor (bFGF) and antibiotics. For the culture of hiPSC colonies 10 ng/ml bFGF was used. The embryoid body (EB) suspensions were maintained in hiPSC medium without bFGF, and the attached EBs were maintained in the same medium with 10% ES cell grade FBS instead of KOSR. All the cell culture reagents were purchased from Life Technologies.

### Generation of lentiviruses and retroviruses

For preparing lentiviruses, HEK293T cells were seeded at a count of 4×10^6^ cells on a 10 cm dish. About 12-16 h later, the cells were transfected with 7 μg of lentiviral expression plasmids, pLenti6/UbC/Slc7a1 (Addgene 17224) (Takahashi et al*.*, 2007) [gift from Shinya Yamanaka, Centre for iPS Cell Research and Application (CiRA), Kyoto, Japan] or pHAGE2-hSTEMCCA (Sommer et al*.*, 2010) (gift from Gustavo Mostoslavsky, Boston University School of Medicine, Boston, MA, USA) along with 3.5 μg of pMD2.G envelope plasmid (Addgene 12259) and 3.5 μg of psPAX2 packaging plasmid (Addgene 12260) [gifts from Didier Trono, École Polytechnique Federale de Lausanne (EPFL), Lausanne, Switzerland], using X-tremeGENE HP (Roche Life Science) following the manufacturer's protocols. For preparing retroviruses, Plate-E cells were seeded at a count of 3.6×10^6^ on a 10 cm dish and 12-16 h later, they were transfected separately with 14 μg of retroviral expression plasmids pMXs-hOCT3/4 (Addgene 17217), pMXs-hSOX2 (Addgene 17218), pMXs-hKLF4 (Addgene 17219), pMXs-c-MYC (Addgene 17220) (Hotta et al*.*, 2009; Takahashi et al*.*, 2007) (gifts from Shinya Yamanaka) and pMXs-mRFP1 (Addgene 21315) (a gift from James Ellis, The University of Toronto, Toronto, Canada). After 24 h, transfection complex containing medium was replaced with fresh medium. The viral supernatant was collected at 48 h, 60 h, and 72 h, pooled and filtered through a 0.45 μm filter. Lentiviral and retroviral supernatants were then concentrated with Lenti-X Concentrator or Retro-X Concentrator (Clontech Laboratories) following the manufacturer's protocols. The concentrated virus was aliquoted and stored at −80°C for future use.

### Human iPSC reprogramming using lentiviral and retroviral vectors

For lentiviral-mediated reprogramming, hADFs were seeded at a count of 3×10^5^ cells on a 12-well plate. About 12-16 h later, the cells were transduced with concentrated hSTEMCCA lentiviruses to get a transduction efficiency of about 70-80% as measured by immunofluorescence analysis of OCT4 expression in the transduced fibroblasts. Six days after transduction, the cells were seeded on mitomycin C-treated SNL feeders at a count of 5×10^5^ on a 6-well plate in the hiPSC medium. For retroviral-mediated reprogramming, hADFs were transduced with lentiviruses to express mouse Slc7a1, and the transduced cells were selected with blasticidin S (Life Technologies, CA). Slc7a1^+^ hADFs were transduced with pMXs-mRFP1 retroviruses, and the RFP^+^ cells were sorted by FACS. For retroviral-mediated reprogramming, about 12-16 h before transduction, RFP^+^ or RFP^−^ Slc7a1^+^ hADFs were seeded at a count of 8×10^5^ cells in fibroblast medium on a 10 cm dish. The cells were subjected to two rounds of transduction with pools of freshly prepared OSKM retroviral supernatants at 1:1:1:1 ratio in an interval of 48 h between the first and the second transductions. On day 4, 5×10^5^ transduced hADFs were seeded on a 6­-well plate containing mitomycin C-treated SNL feeder cells in fibroblast medium. Two days later, the medium was changed to hiPSC medium and the reprogramming cells were fed daily with fresh medium. Cells were harvested on different days for flow cytometry studies and were also observed for the emergence of hiPSC colonies. For the derivation of retroviral hiPSC lines, the RFP^−^ hiPSC clones with hESC-like morphology were picked up 3-4 weeks later, mechanically broken into small clumps and seeded on mitomycin C-treated SNL feeder layer in the hiPSC medium. The clones were subsequently passaged by enzymatic treatment with 1 mg/ml Collagenase IV (Life Technologies).

### Human iPSC reprogramming using episomal vectors

For episomal mediated reprogramming, hADFs were transfected with the Y4 combination of plasmids using Neon transfection system (Life Technologies) based on a protocol previously described (Okita et al*.*, 2011). Briefly, 6×10^5^ hADFs loaded in a 100 μl Neon tip were electroporated with 1 μg each of the plasmids, pCXLE-hOCT3/4-shp53-F (Addgene 27077), pCXLE-hSK (Addgene 27078) and pCXLE-hUL (Addgene 27080) (gifts from Shinya Yamanaka) using the condition, 1650 V/10 mS/3 pulses. Transfected cells were seeded on one well of a 6-well plate in Amniomax (Life Technologies) and fed with fresh medium on alternate days. After 6 days, transfected cells were seeded on mitomycin C-treated SNL feeder cells at a count of 3×10^5^ cells per 10 cm dish in Amniomax and the medium was changed to hiPSC medium on the next day. The hiPSC clones were identified based on morphology, and the hiPSC lines were derived as described in the previous section.

### Human iPSC reprogramming using Sendai virus (SEV) based vectors

For SeV based reprogramming, hADFs were transduced with CytoTune 2.0 Sendai reprogramming vectors (Life Technologies) according to manufacturer's instruction. Briefly, 70-80% confluent hADFs were transduced with 20 μl each of CytoTune 2.0 KOS (hKLF4-OCT4-SOX2), CytoTune 2.0 hc-MYC and CytoTune 2.0 hKlF4 vectors in 1 ml medium per well of a 6-well plate. Following overnight incubation with the reprogramming cocktail, the medium was replaced with fresh fibroblast medium and cells were fed daily with fresh medium. On day 7, the transduced cells were seeded on mitomycin C treated SNL layer in fibroblast medium at a count of 2×10^5^ cells per 10 cm dish. From the next day, cells were fed daily with hiPSC medium. The cells were monitored for the emergence of hiPSC colonies that were subjected to morphology-based isolation.

### Embryoid body mediated differentiation

To initiate EB formation, 4-5­-day-old hiPSCs were harvested as large clumps using 1 mg/ml Collagenase IV and were transferred to a low attachment plate in the EB suspension culture medium. After 8 days of floating culture, EBs formed were seeded on 0.1% gelatin-coated culture dish in the EB attached culture medium to induce further differentiation. The suspension and the attached culture were fed with the fresh medium on alternate days. The attached culture was monitored for differentiation into different cell types for 1-2 weeks. The differentiated cells were analyzed by immunofluorescence assay for the expression of α-fetoprotein (endoderm marker), α-smooth muscle actin (mesoderm marker) and βIII-tubulin (ectoderm marker) to confirm *in vitro* trilineage differentiation potential of hiPSC clones.

### Immunofluorescence assay

To detect the surface markers, reprogramming cells or established hiPSC lines grown on feeders were washed with phosphate-buffered saline (PBS), fixed with 4% paraformaldehyde at room temperature for 20 min and then blocked with a buffer containing 1% bovine serum albumin (BSA) and 5% FBS for 45 min at room temperature. For intracellular protein analysis, hiPSCs and EB differentiated cells were permeabilized with blocking buffer containing 0.1% Triton X-100. Fixed and permeabilized cells were incubated with primary antibodies in the blocking buffer for 4 h at 4°C and 1 h at room temperature followed by incubation with suitably labeled secondary antibodies for 1 h at room temperature. Imaging was done under a fluorescence microscope (DMI6000B, Leica Microsystems, Wetzlar, Germany) with appropriate filters. The primary antibodies used were anti-SSEA-4 (SCR001, EMD Millipore), anti-TRA-1-60 (sc-21705, Santa Cruz Biotechnology), anti-TRA-1-81 (sc-21706, Santa Cruz Biotechnology), anti-NANOG (4903, Cell Signaling Technology), anti-OCT4 (2840, Cell Signaling Technology), anti-SOX2 (3579, Cell Signaling Technology) and anti-LIN28 (3695, Cell Signaling Technology), anti-α-fetoprotein (MAB1368, R&D Systems), anti-α-smooth muscle actin (ab5694, Abcam), anti-βIII-Tubulin antibody (ab5568, Cell Signaling Technology) and the secondary antibodies were anti-mouse or anti-rabbit IgGs conjugated with Alexa Fluor 488 or Alexa Fluor 594 (11029, 11032, 11034, 11037, Life Technologies). All antibodies were used at the dilutions recommended by manufacturers.

### Flow cytometry

A single-cell suspension of reprogramming cells was generated by treating with 1 mg/ml Collagenase IV followed by Accutase (Chemicon Millipore). The cell suspension was filtered through a 70 μm cell strainer (BD Biosciences), centrifuged and then washed with hiPSC medium once. Feeder cells in the sample were depleted using feeder removal micro beads and LD columns on Quadro MACS following the manufacturer's protocol (Miltenyi Biotec). The cells were then stained with labeled antibodies in the hiPSC medium for 30 min in dark at 4°C at recommended dilutions. The antibodies used were CD13-APC (557454, BD Pharmingen), SSEA-4-PE (560128, BD Pharmingen), TRA-1-60-BV421 (562711, BD Pharmingen) and TRA-1-85-AF700 (FAB3195N, R&D Systems) and analyzed using FACSAria III flow cytometer (BD Biosciences). The expression kinetics of CD13, SSEA-4 and TRA-1-60 markers in the cells undergoing reprogramming were analyzed within the cell population expressing pan-human cell marker, TRA-1-85.

### Quantitative real-time PCR

RNA was extracted from flow-sorted cells, hiPSCs and EBs using Tri-reagent (Sigma-Aldrich). 1 μg of total RNA was used for reverse transcription reactions using high-capacity cDNA reverse transcription kit (Life Technologies) according to the manufacturer's instructions. Quantitative RT-PCR was set up with SYBR Premix Ex Taq II (Takara Bio) using specific primers (Table S1) and analyzed with ABI 7500 (Applied Biosystems) or QuantStudio12K Flex (Life Technologies) real-time PCR systems.

### Bisulfite sequencing

Genomic DNA extracted from established hiPSC lines was subjected to bisulfite conversion using EpiTect Bisulfite Kit (Qiagen) according to the manufacturer's recommendations. Using specific primers (Table S1) that can bind bisulfite converted DNA, the promoter regions of human *OCT4* and *NANOG* genes were amplified. The PCR products were cloned into a TA cloning vector pCR2.1 (Life Technologies), and plasmids were extracted from five to ten transformed bacterial clones and were screened by DNA sequencing.

### Teratoma formation

Teratoma assay was performed based on a previously described protocol (Park et al*.*, 2008a). Briefly, the feeder cells were removed from a 4-5-day-old 60-70% confluent hiPSC culture by a brief treatment with 1 mg/ml Collagenase IV, and the cells were harvested using a cell scraper in DMEM-F12 medium. The cell pellet was resuspended in 40 μl of cold DMEM-F12, 80 μl of Collagen I (Life Technologies) and 120 μl of hESC-qualified Matrigel (BD Biosciences) and 100 μl each of this colony suspension was injected intramuscularly into hind limbs of 4-6-month-old immunocompromised B6.CB17-*Prkdc^scid^*^/^SzJ (The Jackson Laboratory) or CB17/Icr-*Prkdc^scid^*/IcrIcoCrl mice (Charles River). The animal experiments were performed following ethical regulations of Christian Medical College, Vellore, India.
